# A new approach to child mental healthcare within general practice

**DOI:** 10.1186/s12875-015-0354-2

**Published:** 2015-10-09

**Authors:** Peter FM Verhaak, Marloes van Dijk, Dick Walstock, Marieke Zwaanswijk

**Affiliations:** NIVEL, Netherlands Institute of Health Services Research, PO Box 1568, 3500BN Utrecht, Netherlands; Department of General Practice, University Groningen, University Medical Center Groningen, Groningen, Netherlands; Medical Center Eudokia, General Practice, Enschede, Netherlands

## Abstract

**Background:**

Child and adolescent mental health problems are frequently not identified and properly treated within general practice. Politicians in the Netherlands are promoting more primary healthcare treatment for mental health problems. The current study aims to evaluate an integrated primary mental healthcare approach for child and adolescent emotional and behavioural problems. This integrated approach allows general practitioners (GPs) to comprehensively explore the request for help, followed by an informed decision to refer, offer short-term treatment within general practice or postpone a decision by asking for additional consultations with youth mental health specialists.

**Method:**

The study is a naturalistic evaluation of Dutch general practices with pre-test and post-test comparison with controls based on data from Electronic Medical Records (EMR). The intervention started in September 2010. EMR data of all GP contacts with children aged 4 to 18 (including diagnosis, prescriptions, referrals) from practices involved in the intervention was used from 1 January 2009 to 31 December 2012. Extra codes were added to the EMR to record aspects of the intervention. Comparable EMR data was used in control practices in 2011.

**Results:**

GPs in the intervention group were able to identify more emotional and behavioural problems after the integrated service had started. They also identified more problems than GPs in the control practices. They were already reluctant to prescribe psychopharmacological medication to children before the intervention, and levels of prescription at intervention GP practices remained low for psychotropic drugs compared to control practices. Referral rates to mental healthcare remained relatively steady after the introduction of the integrated service, but referrals switched from specialized to primary mental healthcare.

**Conclusion:**

An integrated mental healthcare approach within general practice may lead to an increase in detected psychosocial problems among children, and these problems can mainly be treated within the primary care setting.

## Background

Emotional and behavioural problems among children and young adolescents (aged 4 to18) are manifold and prevalent in 14-22 % of the population [1–4]. Such problems are predictors for psychological problems when older [5–9] and early detection and treatment is therefore warranted.

They are frequently not identified within general practice [10, 11] because many young people with mental health problems do not consult their general practitioner (GP) and do not consider GPs an appropriate source of care for mental health problems [12]. Adolescents in particular may be reluctant to seek help for mental health problems [13] and if they do, they mostly express possible concerns in the context of physical problems [14]. Steele et al. [15] and Kramer et al. [16] observed that GPs tend to identify more severe problems while neglecting the more common mental health problems.

As a result, child and adolescent mental health problems are rarely treated in general practice [17, 18]. Many GPs consider themselves unskilled; they are not aware of treatment options for mental health problems [19]. In the Netherlands, responsibility for care for children is compartmentalized and divided between the medical healthcare system and social welfare institutions.

In general, lack of time, competence and access to necessary services combined with an unclear division of tasks between medical and social care are mentioned by GPs as important barriers for providing psychological treatment such as counselling or other psychological interventions [20].

Roberts [21] reports an approach to improving the identification and treatment of youth mental health problems in Australia by strengthening existing primary care services to improve recognition of the bio-psychosocial needs of adolescents. Roberts and Bernhard [19] describe a pilot in which they test this option. Youth mental healthcare is delivered in general practice by a GP, assisted by a nurse practitioner. The GP conducts a thorough bio-psychosocial assessment, gathering collateral information. They produce a “formulation and management plan”; identify appropriate sources of help and support; intervene therapeutically where appropriate and refer if necessary. The number of referrals in the pilot (36 %) was much smaller than in usual practice. The authors conclude that their initiative demonstrates substantial possibilities for general practice in the assessment and management of common mental disorders of children and adolescents.

In the Netherlands, the GP is the first doctor to be contacted when people have concerns about their health and almost all Dutch residents are registered with a general practice. The majority of children and adolescents visit their GP at least once a year [22]. Furthermore, health policy in the Netherlands since 2000 has been directed towards a stronger role for mental healthcare within primary care, especially within general practice. To reach this goal, mental health practice nurses were introduced in 2007.

This reinforcement of primary mental healthcare is mainly focused on mental healthcare for adults. But elements of it, such as the common position of mental health practice nurses within general practice, may help child mental healthcare in general practice as well.

In one pilot area in the Netherlands, an experiment, called the Eureka project, was carried out from 2010 to 2012. This experiment closely resembles the pilot described by Roberts and Bernhard, cited above [19]. The philosophy behind the experiment is the following:

- many aspecific symptoms (such as abdominal pain, obstipation, sleeping problems, behavioural problems that may be self-limiting, etc.) may refer to somatic disease, to problems in the family or to youth mental health issues;
- the GP, who is mostly the professional first contacted, has the initiative to start some kind of treatment;
- to be able to identify and handle child and adolescent mental health problems appropriately, the GP needs time and opportunity for a thorough investment, a network of specialists to refer to and consult for more detailed diagnoses, and options for providing short-term interventions.;
- many of the mental health problems identified may be treated within general practice, provided that general practice has the necessary manpower and know-how available.

The experiment contained a kind of disease management approach, in which GPs got a lump-sum fee for a comprehensive assessment of children (and parents) presumed to have mental health problems (including consultation by specialized consultants) and any further treatment of those problems in general practice. NIVEL, the Netherlands Institute for Health Services Research, carried out an evaluation study based on the electronic medical records (EMR) of the participating practices with data from 2009 to 2012. Research questions were:

1. Has the intervention been carried out as it was designed?
2. Has there been a shift in the identification, treatment and referral of children with psychological or social problems since the start of the Eureka project?
3. Are there any differences in identification, treatment and referral of children with psychological or social problems between the participating GP practices and control GP practices that did not pay extra attention to youth mental health?

## Method

### Setting

The medical centre that carried out the Eureka project consists of four general practices, in which there are six GPs. The GP practices are of average size, ranging from 2459 to 2890 registered patients per practice. At the medical centre, GPs work together with physiotherapists, a midwife, a dietician, a social worker and a psychologist. The medical centre is situated in a deprived area of a large city in the east of the Netherlands.

### Intervention

In September 2010, the medical centre started the Eureka project. Its goal was early identification of child and adolescent psychological or social problems, short-term treatment within primary care where possible, and targeted referral in good time to secondary care as necessary. The project aimed to treat 80 % of children with psychological or social problems at the medical centre. The Eureka project provides facilities to help the GP carry out comprehensive assessments of children and adolescents who are presumed to have mental health problems, consult familiar youth mental health specialists and to provide short-term treatment. The Eureka project consists of the following parts:

- *An extended youth consultation*. When the GP presumes the child has psychological or social problems, the child (and parents) are invited for an extended youth consultation. The extended youth consultation takes 30–45 min in which the problem is further explored by the GP, with help from the school doctor or the Youth and Family Centre as appropriate. Afterwards, the GP decides whether the problem can be treated in primary care or whether a referral is needed. When treatment in primary care is indicated, the GP can bring in the youth mental health practice nurse (YMHPN, see below),
- *The role of the YMHPN*. This practice nurse performs a variety of tasks, such as problem clarification, providing accessible consultation and support for questions/problems concerning raising children, short-term treatment based on cognitive behavioural therapy, psycho-education, educational advice, specific family interventions, supporting the GP with information and advice, and acting as the contact for external parties such as schools and secondary mental healthcare. Preparation of referrals to primary or secondary mental healthcare or youth care is another role of the YMHPN.
- *The role/availability of specialized consultants*. The GPs and YMHPN have the opportunity to contact specialized consultants such as a child psychiatrist, child psychologist or family therapist for advice about diagnosis or treatment. These consultants have been contracted in for the Eureka project.
- *Personal and structured collaboration*. All primary care professionals who are involved in caring for children with psychological or social problems are collaborating at the same medical centre. Because specialized consultants are contracted in on a structural basis, personal and close contact between primary care and specialized mental healthcare is guaranteed as well.

**Fig. 1:**
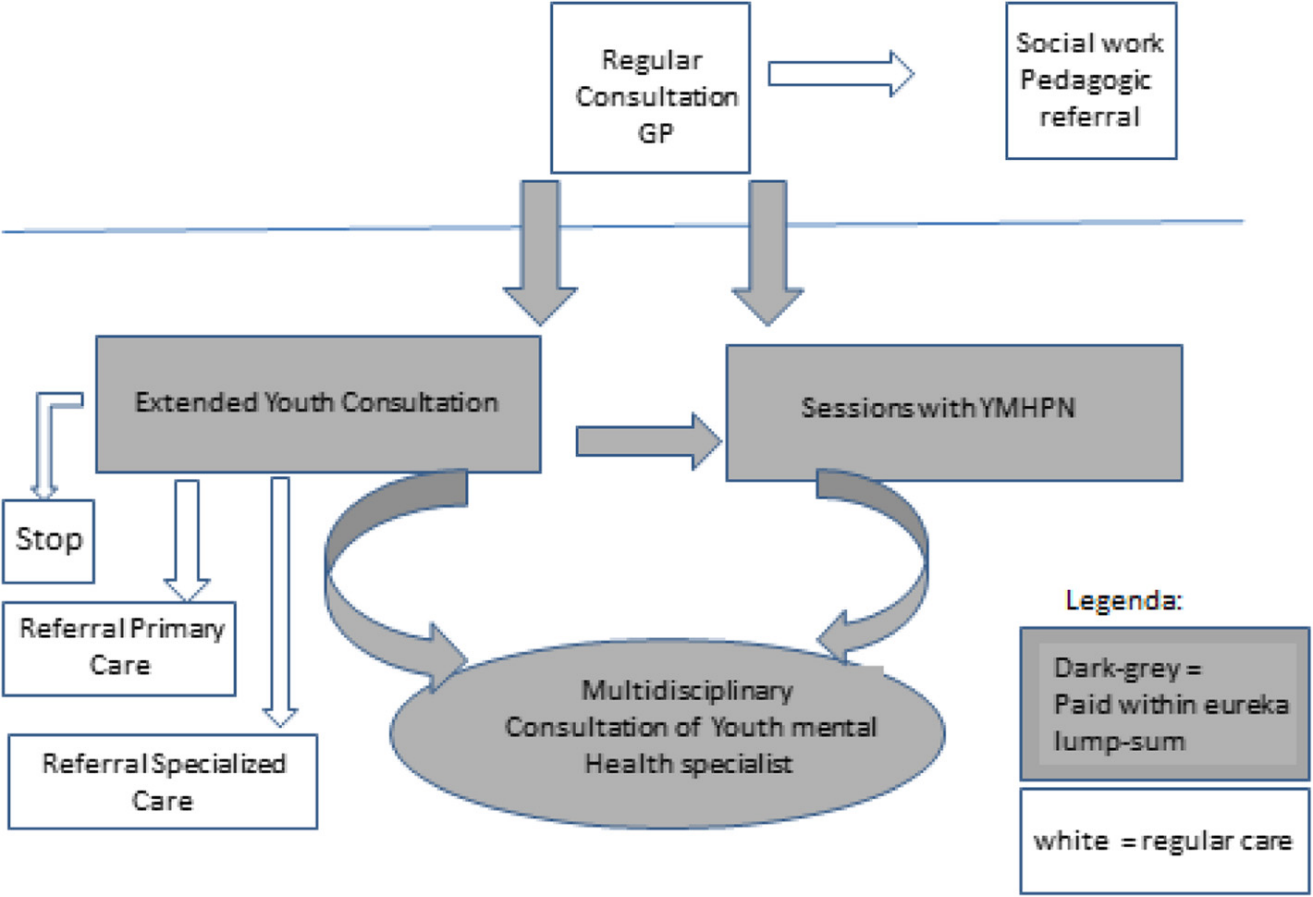
Flow chart of the treatment process within the Eureka project

The routine procedure is as follows: GPs at the Eureka practices see children at their surgeries whom they suspect may have a mental health problem. If they include the child in the Eureka project, they either plan an extended youth consultation or involve the YMHPN directly. The outcome of the extended youth consultation may be an end of the Eureka intervention, further involvement of the YMHPN or referral to pedagogic care, primary youth mental healthcare (both also available in the medical centre) or specialized mental healthcare. Throughout the Eureka intervention, youth mental health specialist consultants may be involved. See Fig. 1 for a flow chart of the treatment process.

#### Funding

Participating practices received a lump sum for each child treated within the Eureka protocol. That reimbursement covers all possible services within the GP practice (GP consultations, use of the YMHPN and any consultations with other specialized consultants). Referrals are not included.

### Target population

The target population consists of children aged between 4 and 18, visiting the GP and in whom the GP suspects psychological or social problems. Approximately 1900 children in this age category are registered with the medical centre.

### Evaluation

We used data from the routine EMRs of GP practices in the medical centre. This system records all diagnoses (as assessed by the GP), treatments, medication prescriptions and referrals. Some modifications were made to the EMRs of the GP practices of the medical centre:

- The YMHPN was able to log in so that the following could be recorded for every consultation: the child’s problem(s), what the YMHPN did during the consultation and the person to whom the child was referred) if applicable).
- Some extra treatment codes relating specifically to the Eureka project were added to the system. GPs and the YMHPN could record e.g. if an extended youth consultation had taken place or if specialized consultants were consulted.

To compare the GP practices at the medical centre against control GP practices, data was derived from routine EMRs from general practices in the NIVEL Primary Care Database (http://www.nivel.nl/en/dossier/nivel-primary-care-database). We used data from GP practices that were comparable with the GP practices at the medical centre involved in the experiment in terms of the level of urbanization and being located in a deprived area. Thirty-three GP practices from urban areas took part, six of which were in deprived areas.

#### Informed consent

Only aggregated, anonymous data have been used, collected via NIVEL Primary Care Database. The NIVEL Primary Care Database is registered with the Dutch Data Protection Authority. The Eureka practices were already part of the NIVEL Primary Care Database or were added to it and its procedures on a temporary basis. All data is collected and handled according to the data protection guidelines of the said Authority. Conforming these guidelines, patients are publicly informed about the participation of the practice in NIVEL Primary Care Database with a possibility to opt out. As Dutch law allows the use of anonymized electronic health records for research purposes under certain conditions, we did not need informed consent or approval by a medical ethics committee for this study (Dutch Civil Law, Article 7:458). The study was approved by the Steering committee of the Netherlands Information Network of General Practice in 2009.

#### Time frame

The period of measurement was 1 January 2009 to 31 December 2012. The Eureka project started in September 2010. To test pre-test versus post-test, data from 2009 was compared with data from 2011. For the comparison between the practices in the experiment and the control practices, data from 2011 was compared between the practices concerned and the control practices.

#### Diagnoses

For the registration of diagnoses, GPs use the International Classification of Primary Care [23]. We analysed the number of children with diagnoses falling under chapters P (Psychological) and Z (Social).

#### Prescriptions

All prescribed medication is registered according to the Anatomical Therapeutic Chemical (ATC) classification.

##### Data analysis

Descriptive analyses were used to calculate the number of children with a psychological or social problem, the number of contacts with these children, and the numbers of prescriptions and referrals. These figures are expressed per 1000 registered patients in the same gender and/or age category. We used t-tests and one-way analyses of variance (ANOVA) to compare the number of children with psychosocial and social problems, treatments, prescriptions and referrals pre-test and post-test. We also compared these numbers for the GP practices at the medical centre concerned and control GP practices. The level of significance was set at *p* < 0.05. Data was analysed with STATA version 13.0.

## Results

### Implementation of the intervention

About one third of the children with a psychological or social problem entered the Eureka programme. For the other two thirds, Eureka was not considered as being indicated; they got usual care from the GP. The Eureka protocol was used for 199 children in the period between October 2010 and December 2012. It has been initiated for 44 of girls (aged 4–18) and for 27 % of boys (aged 4–18) with psychological or social symptoms. Table 1 summarizes the activities carried out within the Eureka protocol.

**Table 1:** Activities executed within the Eureka protocol during 27 months for 199 children

	Number of children	Number of sessions contacts
General practitioners: diagnosis/treatment		
Extended youth consultation	65	68
General practitioners: process		
Consultation GP - psychiatrist	34	37
Consultation GP - family therapist	6	6
YMHPN diagnosis/treatment		
Face-to-face contact with YMHPN	127	471
YMHPN: process		
Registered telephone contacts	117	318
Information request at school	7	9
Report of child abuse	5	6
Multidisciplinary consultation	5	5
Consultation YMHPN – Family therapist	39	47
Consultation YMHPN – Psychiatrist	19	21
Consultation YMHPN – Psychologist	2	2
Consultation YMHPN – General practitioner	45	61
Preparation of referral to primary or secondary care or youth care	20	21

About two thirds of the children who were treated within the Eureka protocol were seen by the YMHPN, for an average nearly four sessions. Sometimes information was exchanged with schools or with an organization against child abuse. The practice nurse had contacts with the child psychiatrist or the family therapist about one third of the children included in the Eureka protocol. One third of those who entered the programme were extensively examined and discussed by the GP within the “extended youth consultation”. The GP consulted a child psychiatrist or a family therapist for about 20 % of the children.

#### Identification of psychosocial problems among 4 to 18-year-olds before and during the intervention

Identification of psychosocial problems within the four practices before the introduction of the Eureka protocol (in 2009) was compared with the identification rate in 2011, when the practices could use the Eureka protocol. Moreover, the identification of children with psychosocial problems in the four practices concerned was compared with comparable control GP practices (Tables 2 and 3).

**Table 2:** Numbers of children with a psychological or social diagnosis, in different gender/age categories, in Eureka practices before and after the start of the Eureka project (per 1,000 visiting patients)

	4 Eureka practices		t-test: pre-test (2009) vs post-test (2011)
Year	2009	2011	
Number of visiting children	1674	1577	
Number of psychosocial diagnoses/1000 patients (4–18 years)	102	134	t = − 4.28 (*p* < .001)
Number of psychosocial diagnoses/1000 boys 4–10 year	144	145	t = −.49 (n.s.)
Number of psychosocial diagnoses/1000 girls 4–10 year	79	93	t = −1.41 (n.s.)
Number of psychosocial diagnoses/1000 boys 11–18 years	81	150	t = −.4.04 (*p* < .001)
Number of psychosocial diagnoses/1000 girls 11–18 years	106	142	t = −2.52 (*p* < .05)

**Table 3:** Numbers of children with a psychological or social diagnosis, in different gender/age categories, in Eureka practices and control practices in 2011 (per 1,000 visiting patients)

	Eureka practices (experiment) (*N* = 4)	Control practices		F-test: Experiment 2011 (A) vs. Control/deprived (B) vs Control/not deprived (C)
		Deprived (*N* = 6)	Not deprived (*N* = 27)	
Year	2011	2011	2011	
Number of visiting children:	1577	1323	10980	
Number of psychosocial diagnoses/1000 patients (4–18 years)	134	42	81	F = 12.63;df =2, 13877; *p* < . 0001 (A > B, C; C > B)
Number of psychosocial diagnoses/1000 boys 4–10 year	145	61	94	F = 1.21; df = 2, 3373; n.s.
Number of psychosocial diagnoses/1000 girls 4–10 year	93	39	56	F = 1.39; df = 2, 3099; n.s.
Number of psychosocial diagnoses/1000 boys 11–18 years	150	36	85	F = 8.2; df = 2, 3629; *p* < .001 (A > B, C)
Number of psychosocial diagnoses/1000 girls 11–18 years	142	30	87	F = 6.74; df = 2, 3767; *p* < .005 (A > B; C > B)

In 2009, 87 children per 1000 aged 4 to18 got a psychological diagnosis and 15 per 1000 a social one. In 2011 these figures were 116/1000 and 19/1000 respectively. In 2011, significantly more children were diagnosed with psychological and social problems by the participating GPs than in 2009, before the start of the Eureka project. In 2011, GPs in the medical centre identified more children with psychosocial problems than GPs in control GP practices. This difference is especially large when compared with control GP practices in deprived areas. Differences between pre-test and post-test and between Eureka practices and control practices are mainly due to an increase of identified problems in the age category 11 to 18.

Table 4 presents the 15 most prevalent psychological and social symptoms and diagnoses for the practices of the Eureka project one year before and one year after the start of the project. It also shows symptoms and diagnoses for control practices from deprived and non-deprived urban areas. The table shows that the increase in identification of psychological symptoms and diagnoses within Eureka practices before and after intervention lies particularly within the categories “other concerns about the child’s behaviour (ICPC P22)” and “anxiety disorder (ICPC P74)”. The largest differences between experiment’s practices and the control practices are in the categories “other concerns about the child’s behaviour” and “parental behaviour (ICPC Z21)”. Control practices in deprived areas record a higher prevalence of “learning problems” than the experiment’s practices do.

**Table 4:** Fifteen most prevalent symptoms and disorders

	Pre-test (2009)		Post-test (2011)		Control (2011)		Control (2011)	
	(*N* = 1674)		(*N* = 1577)		Not deprived area (*N* = 10980)		Deprived area (*N* = 1323)	
	N	N/1000	N	N/1000	N	N/1000	N	N/1000
Psychological symptoms								
Anxious, nervous	6	3.6	8	5.1	49	4.5	5	3.8
Stress	1	.6	1	.6	15	1.4	4	3.0
Depressive feeling	0		6	3.8	47	4.3	2	1.5
Sleeping problems	8	4.8	11	7.0	68	6.2	3	2.3
Eating problems	1	.6	3	1.9	26	2.4	3	2.3
Enuresis	19	11.4	19^2^	12.0^2^	73^2^	6.6^2^	12	9.1
Hyperactive child	26	15.5	33^3^	20.9^3^	172	15.7	9^3^	6.8^3^
Other concerns (psychological) about child/adolescent behaviour	53^1^	31.7^1^	82^1 2 3^	52.0^123^	258^2^	23.5^2^	21^3^	15.9^3^
Learning problems	4	2.4	8^3^	5.1^3^	72	6.6	21^3^	15.9^3^
Other symptoms NEC	3	1.8	9^3^	5.7^3^	34	3.1	1^3^	.8^3^
Psychological disorders								
Anxiety disorder	2^1^	1.2^1^	10^13^	6.3^13^	40	3.6	2^3^	1.5^3^
Depressive disorder	6	3.6	2	1.3	17	1.5	0	
Work/school stress	3	1.8	3	1.9	11	1.0	0	
Social problems								
Parent – child relationship	12	7.2	10^2^	6.3^2^	13^2^	1.2^2^	3	2.3
Parental behaviour	4	2.4	8^2 3^	5.1^2 3^	23^2^	2.1^2^	1^3^	.8^3^

No. of children (aged 4 to 18) with psychological and social symptoms and disorders, recorded one year before the start of the Eureka intervention and one year after the start of the Eureka intervention

^1^significant difference between pre-test and post-test (*p* < .01)

^2^significant difference between post-test and not deprived controls (*p* < .05)

^3^significant difference between post-test and deprived controls (*p* < .05)

Fifty per cent of children with an anxiety disorder entered the Eureka project, 46 of children with “other concerns about the child’s behaviour”, 44 of children with anxiety symptoms (ICPC P01), 29 of children with learning problems (ICPC P24), 25 of children with over-activity (ICPC P21), 22 of children with enuresis (ICPC P12) and 21 % of children with parent–child problems (ICPC Z16).

#### Treatment of psychosocial problems

There was a significant increase in the number of GP consultations per child with a psychosocial problem in the first year after the start of the project (an average of 3.1 consultations per child) compared with the year before the project started (an average of 2.3 consultations per child). In the second year after the start of the project, a child with a psychosocial problem had on average 2.8 consultations with the GP, which was not significantly different than in the period before the start of the project.

In 2011, children with psychosocial problems in the GP practices from the experiment had significantly more consultations with their GP than in children from the control GP practices. Compared to control practices in a deprived area, a child with psychosocial problems in the experiment’s GP practices had an average of 0.99 more GP consultations; compared to control practices that are not located in a deprived area, a child with psychosocial problems from the practices in the experiment had on average 0.65 more GP consultations.

Tables 5 and 6 show the numbers of children with prescriptions for psychotropic medication for the three different periods.

**Table 5:** Numbers of children with a psychological or social diagnosis who received psychopharmacological prescriptions in Eureka practices, before and during intervention

	Eureka practices		t-test: pre-test (2009) vs post-test (2011)
	Before intervention	During intervention	
Year	2009	2011	
Number of children with psychological or social diagnosis	183	253	
Number of children with any psychopharmacological prescription	18 (10 %)	24 (9.5 %)	n.s.
-Analgesics	0	0	n.s.
-Antipsychotics	0	0	n.s.
-Benzodiazepines	2 (1.1 %)	1 (.4 %)	n.s
-SSRIs	1 (.6 %)	2 (.8 %)	n.s
-Psychostimulants	11 6.0 %)	17 (6.7 %)	n.s.
-Other	4 (2.2 %)	5 (2.0 %)	n.s.

*n.s* not significant

**Table 6:** Numbers of children with a psychological or social diagnosis who received psychopharmacological prescriptions in Eureka practices and control practices in 2011

	Eureka practices (*N* = 4)	Control practices		F-test: experiment 2011 (A) vs. control/deprived (B) vs control /not deprived (C)
	During intervention	Deprived (*N* = 6)	Not deprived (*N* = 27)	
Year	2011	2011	2011	
Number of children with psychological or social diagnosis	253	133	1361	
Number of children with any psychopharmacological prescription	24 (9.5 %)	7 (5.3 %)	228 (16.8 %)	F = 9.76 (df = 2, 1744), *p* < .001) (C > A,B)
-Analgesics	0	0	2 (.2 %)	n.s.
-Antipsychotics	0	2 (1.5 %)	23 (1.7 %)	n.s.
-Benzodiazepines	1 (.4 %)	1 (.8 %)	11 (.8 %)	n.s.
-SSRIs	2 (.8 %)	0	7 (.5 %)	n.s.
-Psychostimulants	17 (6.7 %)	3 (2.3 %)	145 (10.7 %)	F = 6.32; df = 2, 1744; *p* < .005 (C > B)
-Other	5 (2.0 %)	1 (.8 %)	72 (5.3 %)	F = 5.09; df = 2, 1744; *p* < .01 (C > B)

Although there was an increase in the number of children with identified psychosocial problems in the first year after the start of the Eureka project , the number of prescriptions remained low: significantly lower than in control practices, although not lower than in control practices in deprived neighbourhoods. In control practices from non-deprived areas, more prescriptions for psychostimulants were observed.

#### Referrals of children with psychosocial problems

**Fig. 2:**
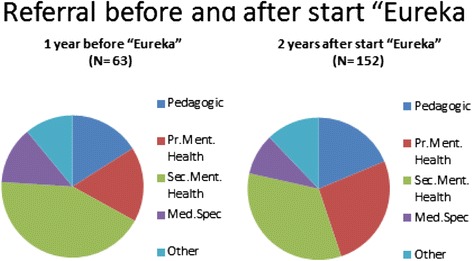
Youth mental health referrals before and after start of Eureka project

If the psychosocial problems of the child were regarded as too severe to treat at the medical centre, GPs referred the cases to facilities outside the medical centre. The proportion of children referred hardly changed after the start of the Eureka project. In the year before the start of the project, 63 out of 216 children with a psychosocial diagnosis (29) were referred, compared to 65 out of 250 (30) in the year after the start of the project and 68 out of 267 (20) a year later. Referrals to mental healthcare (primary mental healthcare and specialized mental healthcare combined) involved 38 (18), 40 (16) and 51 (19) respectively of children with a psychosocial diagnosis. The specific settings to which children with psychosocial problems were referred to showed some changes (Fig. 2).

In each period, most children were referred to secondary mental healthcare, but after the start of the Eureka project, relatively more children were referred to primary mental healthcare. The number of referrals to medical specialists decreased after the start of the Eureka project.

In 2011, when the Eureka project was running, practices referred relatively few children, compared to control practices in deprived areas: 24 % of all children with psychosocial diagnoses from the Eureka practices, compared to 50 % of the children with a psychosocial problem in practices in deprived areas. On the other hand, control practices not located in deprived areas referred even fewer, namely 18 % of children with psychosocial diagnoses.

## Discussion

We studied an intervention that allows GPs to comprehensively assess child and adolescent psychological and social problems, and that provides opportunities for short-term treatment or quick, focused referral. The intervention led to an increased identification rate for psychological and social problems, more GP contacts because of such problems, a restricted psychopharmacological approach and a small change in the emphasis of referral destinations (from secondary care to primary mental healthcare).

In a representative Dutch study conducted in 2008 in 82 general practices [24] with a registered population of 73,432 patients aged 0 to 18, GPs recorded psychological symptoms and diagnoses in 6.9 % of children and adolescents. Psychotropic medication was prescribed to 15 of those aged 0 to 12 and 29 % of those aged 13–18. The figures for referrals to primary and secondary child mental healthcare showed an increasing trend from 2004 – 2008 from 14 % to 19 % (for 0 to 12-year-olds) and from 18 % to 23 % (for 13 to 18-year-olds).

When we compare the Eureka project with this Dutch representative study, we find higher recognition rates, lower prescription rates and a referral rate to primary mental healthcare and specialized mental healthcare for Eureka that is lower than the average in the Netherlands. In this respect the aim of the project to recognize and treat more child mental health problems within general practice is fulfilled.

Compared to control practices, the Eureka intervention appears to be especially directed at specific behavioural problems, anxiety and relational problems with parents. Differences with control practices in deprived areas were larger than with control practices in urban settings that were not deprived, except for “learning problems”. This is more prevalent in control practices in deprived areas than in other control practices and in the Eureka practices. It is possible that help-seeking behaviour by parents in deprived areas for their children’s problems is less prominent than in non-deprived areas, but schools may be playing an important role when people seek help for learning problems.

The question may arise if the lump fee, to be received for any patient included in the Eureka protocol, may not act as a reward to identify more possible mental health problems. This study cannot answer that question. It should be possible, however, to account for the several modules of the Eureka intervention, actually used, afterwards and calculate with insurers a reasonable lump fee on a yearly basis.

These results show that the Eureka protocol may contribute to the early detection and treatment of psychosocial problems among children and adolescents. It provides general practices with opportunities to spend more time on and pay more attention to psychosocial problems with relatively mild symptoms. The increased detection rate was not accompanied by an increase in psychopharmacological treatments, nor by fewer children being referred to more specialized treatment. In this respect, there was a modest switch from specialized to primary mental healthcare. This may be attributable to the fact that more specific and specialized competencies were added to the general practice by the deployment of the YMHPN and by the frequent consultations with the psychiatrist, family therapist and psychologist. The Eureka protocol has an impact on all the barriers to psychological treatment in general practice that were mentioned in the introduction.

Our study design was exploratory. Eureka has been implemented by GPs who also developed the project, so selection bias is plausible. Nevertheless, no significant differences could be found in the identification of psychosocial diagnoses before Eureka compared with the control practices, suggesting that GPs in the medical centre did not tend to identify more psychosocial problems before the start of the project.

Our study was a naturalistic study in which the Eureka intervention should be considered as a “black box”. Contrary to controlled randomly designed effect studies, we were only able to analyse routinely collected data before and after the implementation of the intervention and compare it with data routinely collected elsewhere. We cannot therefore report on the integrity of the intervention as a whole or the standardization of the assessments and therapies used.

Given the exploratory character of the study and the lack of valid outcome measures, no conclusions can be drawn about the effectivity or cost-effectiveness of the project.

Regarding our measurements, we should be aware that the use of routinely collected data has both advantages (no extra effort for GPs, no “experimenter” effect or social desirability) and disadvantages (no extra checks on reliability; only data that is already available can be used).

## Conclusion

We conclude that Eureka, when applied by motivated GPs, may lead to an increase in detection of children’s psychosocial problems that can be treated largely within the primary care setting. The comprehensive approach with consultations by experts and the using a youth mental health nurse has been proven to be feasible. It is not to be expected that referrals will decrease, as the project itself already increases the number of identified cases. Whether this extra assistance within general practice will be cost-effective in the long run should be the aim of a subsequent study.

## Abbreviations

ATC: Anatomical therapeutic chemical (classification of prescriptions)
EMR: Electronic medical record
GP: General practitioner
ICPC: International classification for primary care
YMHPN: Youth mental health practice nurse
